# Ten-year follow-up of giant basilar aneurysm treated by sole stenting technique: a case report

**DOI:** 10.1186/1752-1947-4-64

**Published:** 2010-02-22

**Authors:** Marco Zenteno, Camilo R Gómez, JA Santos-Franco, Fernando Vinuela, Y Aburto-Murrieta, Angel Lee

**Affiliations:** 1 Departamento de Terapia Endovascular Neurológica, Instituto Nacional de Neurología y Neurocirugía, Universidad Nacional Autónoma de México, Av. Insurgentes Sur 3877, 14269 Mexico City, Mexico; 2 Division of Critical Care Neurology, Alabama Neurological Institute, Birmingham, AL 35209, USA; 3 Department of Neurosurgery, Centro Médico Nacional "La Raza", IMSS, Mexico City, Mexico; 4 Department of Radiological Sciences, David Geffen School of Medicine at UCLA, Los Angeles, CA 90007, USA; 5 Department of Neurological Endovascular Therapy, Instituto Nacional de Neurologia y Neurocirugía, Mexico City, Mexico; 6 Stroke Unit, Hospital Ángeles del Pedregal, Department of Neurosurgery, Instituto Nacional de Ciencias Médicas y Nutrición Salvador Zubirán, Mexico City, Mexico

## Abstract

**Introduction:**

The sole stenting technique has emerged as a new tool for the management of intracranial aneurysms. However, several concerns have emerged about the long-term behavior of intracranial stents, particularly their safety and efficacy.

**Case presentation:**

We present the first case of an intracranial aneurysm intentionally treated with the sole stenting technique. After ten years of clinical and imaging follow-up, the lesion has healed and no intrastent stenosis is observed.

Several issues concerning this technique are discussed. For instance, the modification of the angle and intra-aneurysmal thrombosis may account as positive effects; negative outcomes include in-stent thrombosis or stenosis.

**Conclusions:**

This case report, involving a long clinical and imaging follow-up, provides an example of the effectiveness, safety, durability and simplicity of the sole stenting technique in the management of intracranial aneurysms.

## Introduction

Giant aneurysms of the posterior circulation are uncommon. However, they have a high incidence of morbidity and mortality. The sole stenting of intracranial aneurysms has been recently introduced in the armamentarium of their management [1,2] and is backed up by experimental and clinical studies [3-5]. This suggests that the intra-aneurysmal flow is shifted by the placement of a stent within the parent vessel.

## Case presentation

**Figure 1:**
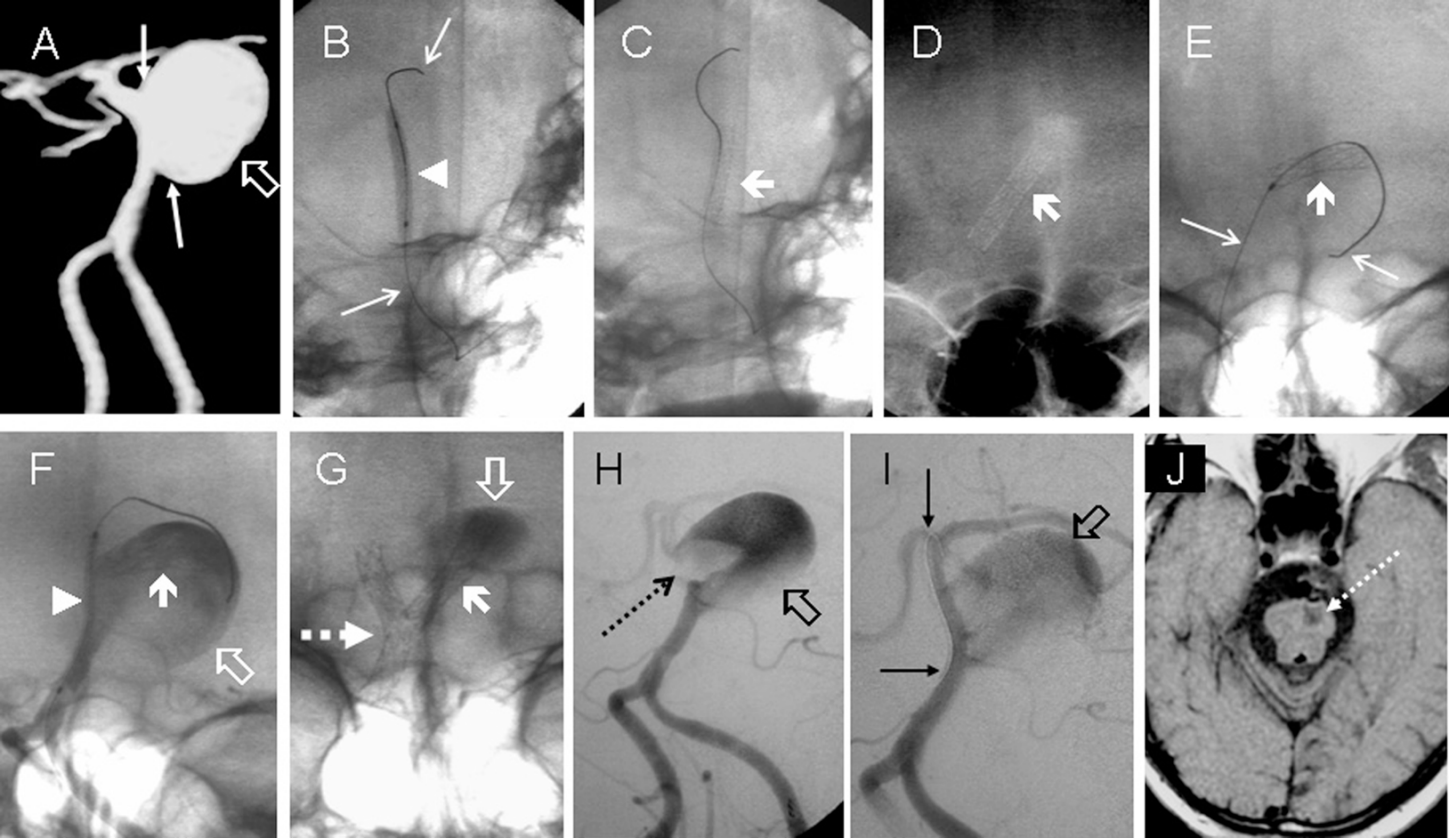
**Imaging of the aneurysm before (A), during the stenting (B-G) and in vascular rescue procedure (H-J)**. A) Preoperative angio-CT: Aneurysm of the basilar artery (hollow arrow) and evidence of wide neck (thin arrows). B) to G) First procedure. A balloon-expandable stent (arrowhead in B and thick arrow in C) is advanced on a microguidewire (thin arrows in B). Once the stent was deployed, it slid out of place towards the sac (thick arrows in D). The whole misplaced stent was gently pushed into the sac (thick arrow in E and F) using a balloon mounted on the same microguidewire (thin arrows in E). A second balloon-expandable stent (arrowhead in F) is placed over the entire length of this wide-necked aneurysm (hollow arrow in F). This second balloon-expandable stent is in a correct position within the parent vessel (dotted arrow in G), the first misplaced BES lies within the aneurysm (thick arrow in G) and a sluggish intraaneurysmal vortex motion is clearly shown (hollow arrow in G). Second procedure. Neurological impairment four hours later explained by in-stent thrombosis. An intravascular clot initially emerging from the thrombosing sac (hollow arrow in H) overflows into the basilar artery (dotted arrow in H). Both pharmacological and mechanical measures using a microguidewire (thin arrows in J) successfully recanalize the arterial tree distal to the aneurysm (J). Imaging follow-up. At one week, the T1 magnetic resonance imaging shows a residual stroke in the ventral portion of the cerebral peduncle (dotted arrow in K).

A 43-year-old Mexican Hispanic man arrived in our hospital with a chronic headache. His neurological examination was unremarkable. The imaging workup showed a giant basilar artery aneurysm, dimensions 32 mm × 20 mm (Figure 1A), and that the lesion was located at an angled portion of the vessel. For the management of this aneurysm, we decided to place a stent at the aneurysm neck in order to: 1) straighten the shape of the parent vessel; 2) promote a shift in the pattern of the inflow into the sac and the outflow from the lesion; and 3) foster the thrombosis of the aneurysm.

The lesion was reached through a left vertebral approach with a 6F guiding catheter (Envoy, Cordis, Miami Lakes, FL). The medical periprocedural management at that time (1997) was empirical, as no rules for anticoagulation or antiplatelet treatment had been defined for intracranial stents. The vascular neurologist advised that the patient be placed under full anticoagulation without any antiplatelet drug. A 3.5 mm × 12 mm balloon-expandable stent (BES) (AVE Medtronic; Advanced Vascular Engineering, Santa Rosa, CA) was advanced and deployed at the neck of the lesion (Figures 1B, C).

When trying to retrieve the balloon, the stent slipped backwards and its distal end tilted towards the sac. (Figures 1C, D). We therefore decided to carefully slip the stent forward into the aneurysm by inflating the balloon again and pushing the stent distally until the whole of it was floating within the aneurysm (Figures 1E, F). A 3.5 mm × 18 mm BES was advanced into the lesion and subsequently deployed within the parent artery to cover the full length of the diseased portion of the vessel, including the aneurysm neck (Figure 1G) In the immediate post-procedural angiographic runs, we observed a sluggish intra-aneurysmal vortex motion and a correction of the shape of the parent vessel to a 45-degree angle was conducted. The neurological status of the patient was unchanged and he was then discharged to the neurosurgical ward.

A control digital subtraction angiography (DSA) performed four hours later to counter clinical deterioration showed thrombosis of the aneurysm along the basilar artery (Figure 1H). Rapid recanalization of the vessel was obtained through a mechanical procedure (balloon angioplasty) with the use of antiplatelet treatment (ticlopidine 250 mg and aspirin 100 mg) (Figure 1I). The patient subsequently recovered and this episode left a pontine stroke as a sequela (Figure 1J).

**Figure 2:**
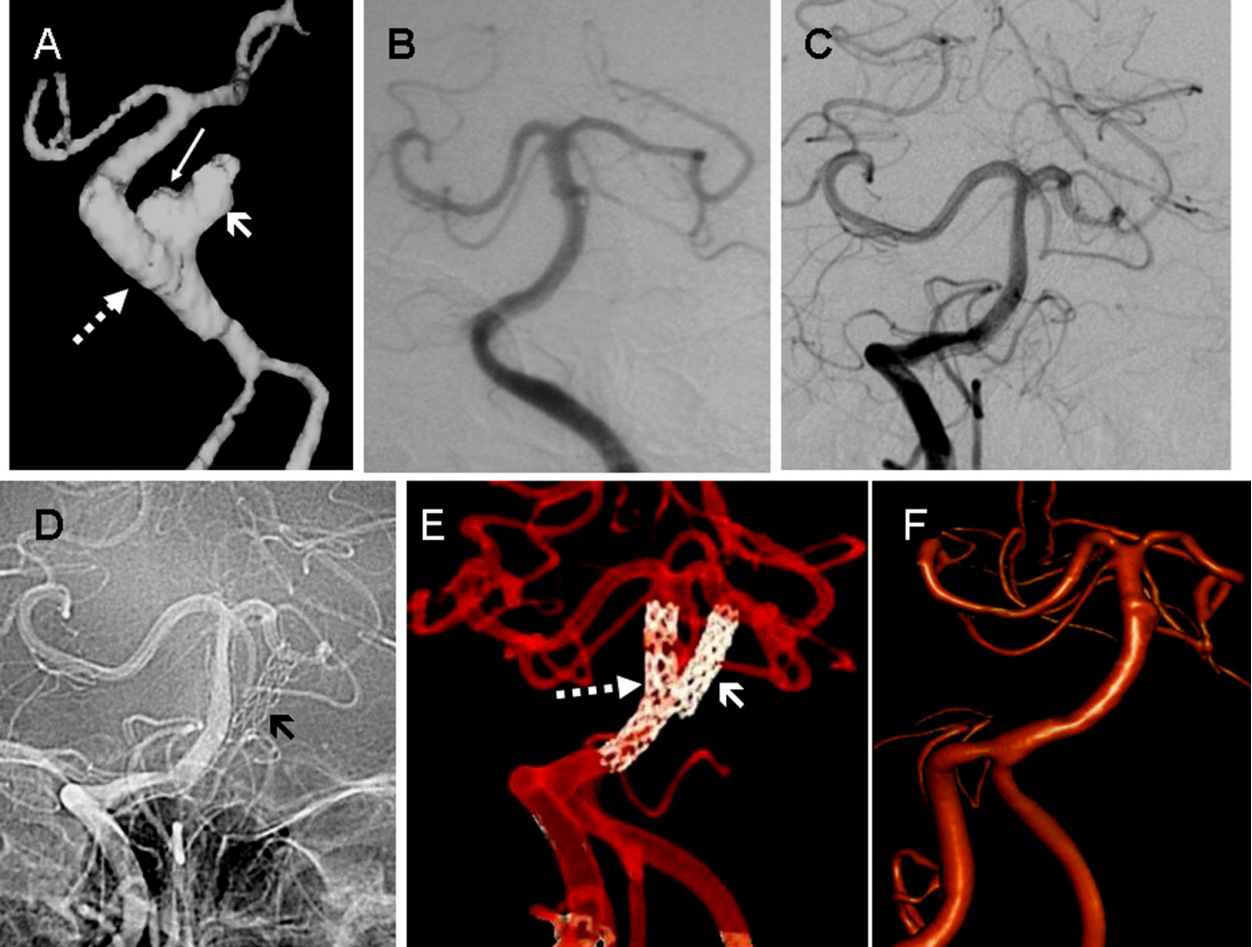
**Imaging follow-up spanning ten years**. A) Imaging at three months. Angio-CT reconstruction shows the first (thick arrow) and the second (dotted arrow) with a dog-ear residual sac (thin arrow). B) One year imaging. Digital Subtraction Angiography showing complete healing of the aneurysm (B). No in-stent stenosis. No recanalization. C) to F): Ten years imaging. Control Digital Subtraction Angiography runs (C and D) show no aneurysm and a patent stented vessel. The misplaced intra-aneurysmal extravascular stent is shown as well (thin arrow in D). Three-dimensional reconstruction (E and F) confirms these findings, with a nice visualization of both stents in dual reconstruction (dotted and thick arrows in E). No in-stent stenosis. No recanalization.

At ten years after the operation, the patient has a minor paresis, with a modified Rankin Scale of 2. A new imaging follow-up with DSA and three-dimensional reconstruction has been performed, which showed that the aneurysm remains occluded (no recanalization), the parent vessel is still patent, and no in-stent stenosis is present (Figure 2C to 2F). The clinical evolution of the patient is described in Table 1.

## Discussion

Complex aneurysms of the posterior circulation are uncommon. Nevertheless, their surgical and endovascular management represents a challenge because their incidences of morbidity and mortality are high. Giant aneurysms are dynamic lesions with intraluminal thrombus formation, occasionally giving rise to thromboembolic events [6]. Other methods have also been described, such as medical management with anticoagulants and surgical options like by-pass surgery [6,7], and interventional procedures have become widely accepted and stand as the first option in many cases, especially in posterior circulation giant aneurysms.

The use of stents as an ancillary tool in the management of wide neck intracranial aneurysms with coils started in 1997. The technique aims to build an artificial neck to the aneurysm, thus preventing the occlusion of the parent vessels by coils bulging into the lumen [8]. Furthermore, the stent promotes the redirection of the flow away from the aneurysm, increasing the rate of complete embolization and decreasing the rate of recanalization [8,9]. Both in vitro and computerized models of aneurysms treated by stent placement without coiling have shown that this device promotes the redirection of the bloodstream away from the aneurysm, which produces a sluggish intra-aneurysmal vortex motion with subsequent blood stasis [10]. In animal models, the stents have shown their ability to induce the permanent occlusion of the aneurysms due to an intra-aneurysmal thrombosis, which may be explained by the aforementioned hemodynamical modifications. Another thrombosis-inducing effect is the ability to change the flow pattern within the aneurysmal sac from a helical into a noncoherent turbulence capable of inducing thrombosis [3,11]. Additionally, after the aneurysm has thrombosed, the luminar surface of the stent is covered with neointima and the treated arterial segment is remodeled.

The sole stenting technique has also been applied in humans. Our initial experience in a prospective series of 20 cases has yielded encouraging results [3], as this technique promotes the healing of the aneurysm and provides a certain protection against further rupture. We have stressed the safety and the effectiveness of this technique in posterior circulation aneurysms [3,12]. Still, the placement of an intracranial stent encompasses some possible complications. However, the development of new devices, such as BES and self-expandable stents (SES), has decreased the incidence of technical complications like arterial dissection [5,9]. The acute thrombosis of the device is also highly uncommon due to routinely administered antiplatelet medication in any stent deployment.

In our case, the in-stent thrombosis occurred due to an inadequate antiplatelet regime [1,3]. Han *et al*. have reported the case of a patient with in-stent thrombosis in a series of 10 complex aneurysms treated by stent-assisted coiling (BES) [14]. In a series of 48 intracranial aneurysms managed with SES, thromboembolic events occurred in a total of six cases, of which four happened when the mesh was crossing the stent with the coils [9]. An intrarterial or intravenous infusion of a glycoprotein IIb-IIIa inhibitor (abciximab) resolved this complication in most of cases. In a series of 74 aneurysms, most of them treated by stent-supported coiling, Fiorella et al. reported six symptomatic thromboembolic complications, wherein one of them had a late in-stent thrombosis by discontinuation of antiplatelet drugs [8]. On the other hand, in our prospective series of 20 complex aneurysms of the posterior circulation managed by the sole stenting technique, we encountered no intrastent thrombosis [3]. However, in analyzing our series of more than 200 aneurysms treated using an intracranial stent (either sole stenting or stent-assisted coiling), a thrombosis within the stent has been observed in about 10% of cases, all of them using SES. This complication has never occurred with a BES, except in the case presented in this report. As opposed to Fiorella et al. [8], we think that the low radial force of the SES may lead to poor wall apposition of the stent, which may cause a thrombogenic process.

In order to avoid in-stent acute thrombosis we designed a protocol that has been recently published and discussed [15]. This protocol pertains to the following: 1) In unruptured aneurysms, either a double regime (clopidogrel, 75 mg qd and acetylsalicylic acid, 100 mg qd) is started at least four days before the procedure, or a loading dose of 300 mg of clopidogrel is given four hours before the procedure; 2) In ruptured aneurysms, intravenous antiplatelet regime is started during the deployment of the stent. In all cases, the patient is kept under antiplatelet drugs using oral medication (clopidogrel, 75 mg qd and acetylsalicylic acid, 100 mg qd) and one additional drug (usually aspirin) for the next six months. When a neurosurgical procedure is foreseeable, we suggest: a) If hydrocephalus shunting or hematoma removal is likely, the patient should be excluded from a stenting procedure to avoid perioperative bleeding; b) If moderate hydrocephalus is present with Fisher III-IV, either do not to place a stent at all or place the shunt before the stent; c) When the likelihood of being operated is remote but is still probable, another option is to keep the patient on an intravenous antiplatelet regime (tirofiban) for three days. In case necessary, the discontinuation of tirofiban will allow a surgery four hours thereafter. This is not the case with oral antiplatelet drugs (clopidogrel and ticlopidine act on ADP and aspirin act on thromboxane-A2). Both of them require seven days of discontinued use of abciximab (which acts on fibrinogen cross-linking) and 48 hours of discontinued administration of antiplatelet drugs in order to ensure a safe surgery.

Another feared complication after intravascular stenting is the development of intimal hyperplasia leading to an in-stent stenosis. In coronary stenting for atherosclerosis, this occurs in up to 50% of reported cases. In intracranial atherosclerosis, the rate of restenosis may be present in up to 40% of cases [16]. Nonetheless, the use of intracranial stents in aneurysm raises a different issue: Han et al. found no stenosis in a series of 10 cases treated with a BES [14], and Lylyk et al. found no in-stent stenosis in 29 cases (SES) during a median follow-up period of 7.3 months [9,17]. In another series, Fiorella et al. reported an in-stent stenosis in 3.8% of 74 treated cases of aneurysms (SES) [8]. In this article, the authors state that, although the true incidence of clinically relevant in-stent stenosis after Neuroform stenting would not be clear until a larger volume of follow-up data become available, the lack of any similar reports would suggest that this occurrence is likely to be relatively rare. In our series, we encountered only one case of lumen reduction due to intimal hyperplasia (the patient had a severe dyslipidemia). This finding was asymptomatic and healed at 12 months under medical treatment (statins) [3]. We are not oblivious to the fact that this patient is only one case. However, this case is highly significant by its unique ten-year follow-up and shows that intracranial stenting may be safe and efficacious in the long run.

## Conclusions

To our knowledge, this is the first case where the sole stenting technique was employed. Even if the presence of another stent within the aneurysm might have favored the thrombosis of the lesion (foreign body), both the correction of the angle and the hemodynamical changes were present right from the beginning of the whole process. The intraaneurysmal coils act as a foreign body. However, they do not in any way prevent recanalization. Even if ours is only one case, this exceptionally prolonged reported follow-up of ten years provides a superb and palpable example of the effectiveness, safety, durability and simplicity of the sole stenting in the management of intracranial aneurysms, preventing the recanalization of the lesion without the alleged long-term danger of in-stent restenosis.

## Abbreviations

BES: balloon-expandable stent; DSA: digital subtraction angiography; SES: self-expandable stents; ADP: adenosine diphosphate.

## Competing interests

The authors declare that they have no competing interests.

## Authors' contributions

ZM and GC devised the strategy of treatment, ZM, GC and LA treated the patient, ZM, LA and SF made the follow-up of the patient, designed, planned the manuscript and drafted the initial version, VF and AM revised, corrected the manuscript and performed the bibliographic search, ZM, GC, SF, VF, AM and LA read and approved the final manuscript.

## Consent

Written informed consent was obtained from the patient for publication of this case report and any accompanying images. A copy of the written consent is available for review by the Editor-in-Chief of this journal.
